# Multi-label spacecraft electrical signal classification method based on DBN and random forest

**DOI:** 10.1371/journal.pone.0176614

**Published:** 2017-05-09

**Authors:** Ke Li, Nan Yu, Pengfei Li, Shimin Song, Yalei Wu, Yang Li, Meng Liu

**Affiliations:** 1 Ergonomics and Environment Control Laboratory, Beihang University, Beijing, China; 2 China Academy of Space Technology, Beijing, China; 3 School of Automation Science and Electrical Engineering, Beihang University, Beijing, China; Jiangnan University, CHINA

## Abstract

In spacecraft electrical signal characteristic data, there exists a large amount of data with high-dimensional features, a high computational complexity degree, and a low rate of identification problems, which causes great difficulty in fault diagnosis of spacecraft electronic load systems. This paper proposes a feature extraction method that is based on deep belief networks (DBN) and a classification method that is based on the random forest (RF) algorithm; The proposed algorithm mainly employs a multi-layer neural network to reduce the dimension of the original data, and then, classification is applied. Firstly, we use the method of wavelet denoising, which was used to pre-process the data. Secondly, the deep belief network is used to reduce the feature dimension and improve the rate of classification for the electrical characteristics data. Finally, we used the random forest algorithm to classify the data and comparing it with other algorithms. The experimental results show that compared with other algorithms, the proposed method shows excellent performance in terms of accuracy, computational efficiency, and stability in addressing spacecraft electrical signal data.

## I. Introduction

After the spacecraft steps into the orbit flight phase, it is fully working in a high vacuum, cold black and strong solar radiation environment. When a spacecraft launches, it is impossible perform maintenance while in orbit, and thus, it is necessary to perform diagnostics and forecasting for possible faults [1]. Spacecraft electronic load systems are typically non-linear time-dependent systems, and the coupling of the internal components is highly nonlinear, which implies that it is complex and uncertain. Mutations of the internal load signals occur frequently, and when a fault occurs in the system, the cause of the accident will be intertwined. If there is no reliable source of information and no suitable analysis methods, and we are left to rely solely on assumptions and speculation, it is difficult to determine the exact cause of a fault [2, 3]. It is also difficult to identify a fault because of the complexity of the spacecraft electronic load system. We are still dependent on the experience and knowledge of experts on the diagnosing of spacecraft faults, however experts in different fields give different judgments for the same fault. Thus, it is not easy to meet the spacecraft fault detection in real time with multi-label classification. If we store the experience of different experts into a knowledge base, which is based on a pattern recognition algorithm, we then can realize an improvement in the real-time nature of the operations and in the efficiency of the spacecraft electronic load system fault detection [4].

Identification and analysis of spacecraft testing data are processes that involves feature extraction and classification of the collected signal waveform data. Spacecraft testing data variations mainly include slowly varying data, mutation data, and periodic variation data. There are some multi-label classification problems that must be solved urgently, such as having a large amount of testing data, high feature dimensions, high computational complexity, and a low recognition rate in the identification process of the spacecraft electrical characteristics monitoring system.

In a previous study, for example, Liu Y uses off-line fuzzy clustering and an online support vector machine to recognize the spacecraft electrical data and uses the weighted proximal support vector machine for the classification and recognition of the electrical data. In the process of recognition, the PCA feature method is used to reduce the dimension of the data, and the results of the classification are better. However, there are fewer sample set types, the amount of data is small, and the classification accuracy is not high [5, 6, 7, 8].

Relative to other algorithms, the random forest algorithm has a large advantage when analysing many of the data sets. It can address high-dimensional data with a good ability to learn from a large amount of data, and it can realize learning and classification for nonlinear sample data. It has a unique advantage in the identification of electrical characteristic signals. In the actual diagnosis process, if the input of the electrical characteristic data is too large, the training complexity will greatly increase. For complex and high-dimensional feature systems, a large amount of data will affect the training and classification efficiency, and it leads to a decline in the recognition accuracy rate. How to obtain the sensitive features from the high-dimension characteristics becomes one of the bottlenecks for the fast and accurate identification of electrical signals [9, 10, 11, 12]. The deep belief network (DBN) has an excellent learning ability by means of using a multi-layer neural network. The learning characteristics that are achieved by the DBN include providing more of the essential features of the original data. In addition, the DBN algorithm can overcome the gradient diffusion problem, especially when the gradient descent method is trained by using a layer by layer initialization method from a multilayer neural network [13].

The remainder of this paper is organized as follows. In section II, the schematic of the system is introduced. The methodology of the feature dimension reduction and the random forest algorithm is presented in section III. The experimental results show that the proposed approach can achieve high classification accuracy for multi-class spacecraft signals, compared with the conventional classification methods discussed in Section IV. Final conclusions are given in Section V.

## II. The identification system

**Fig 1:**
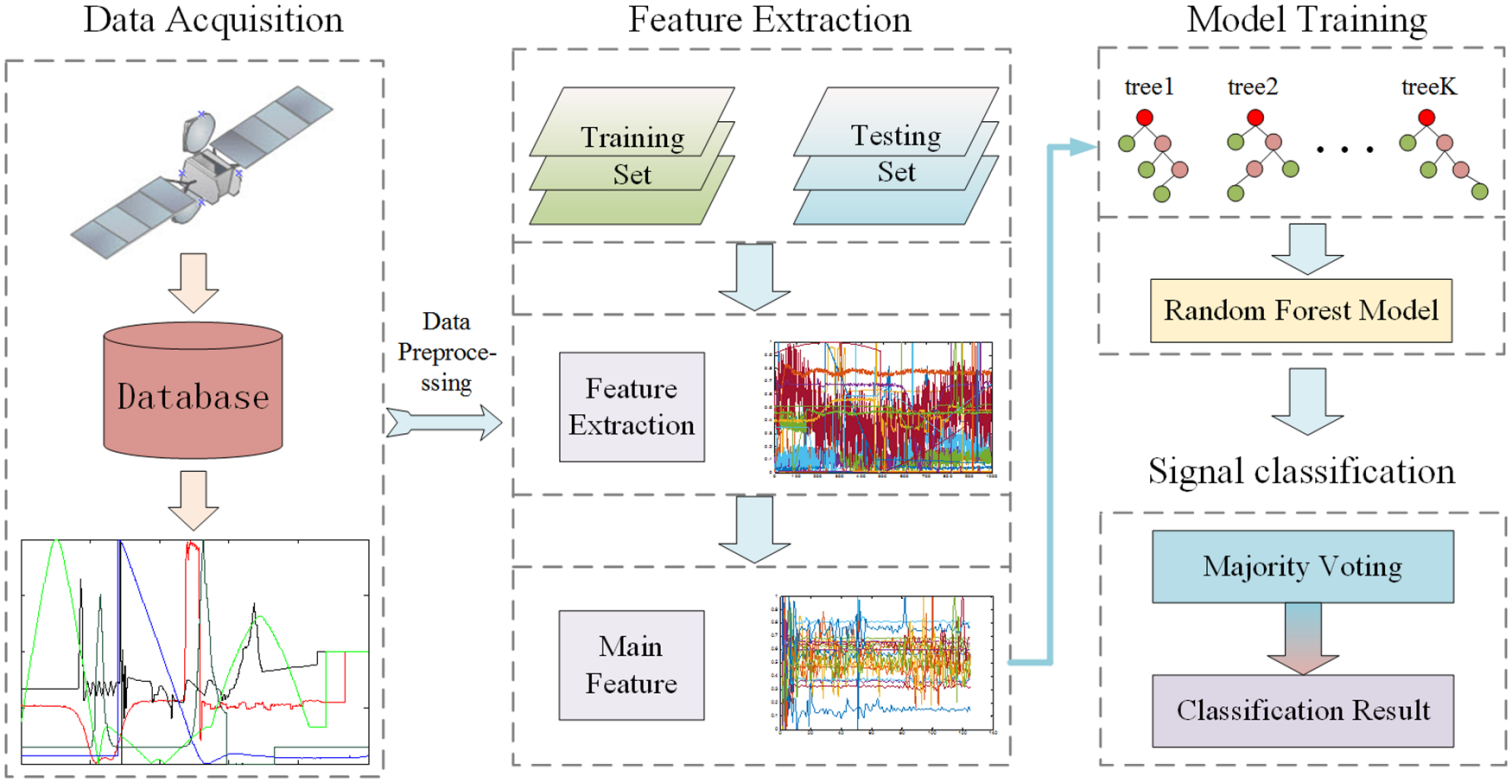
The flow chart of algorithm by this article. The process of the algorithm model consists of three parts: data acquisition using wavelet denoising, feature extraction using DNN method, and signal recognition using random forest. The flow chart of the typical test data sample and a typical standard data sample obtained from the electrical characteristics of the spacecraft are shown in the Fig.

In this paper, the process of the algorithm model consists of three parts: data acquisition using wavelet denoising, feature extraction using DNN method, and signal recognition using random forest. After collecting and preprocessing the data, we use a deep neural network to extract the feature vectors from the training and testing sets. Then, the random forest algorithm classification model is trained by the training set, which means that we can use the testing set for validation. Finally, we obtain the classification results. Specifically, the spacecraft is detected using a total of 50 channels in the data, and data from each channel are collected at a rate of more than 30 MB/S. The flow chart of the algorithm is shown in Fig 1.

## III Feature extraction and classification algorithm

### A. wavelet denoising

In the process of signal recognition and classification, the collected data could contain a large amount of noise, and to improve the accuracy of the classification and recognition, a digital filter is essential.

In this paper, the wavelet threshold denoising method is used for data processing [14, 15]. The wavelet threshold denoising method is a process in which wavelet decomposition is used to separate the original signal, dividing it into components of high frequency and low frequency, and then, when applying the reconstruction process, there is a better effect of the mutant noise.

Define each section of the spacecraft electrical characteristics data as vectors in the form of

$$\begin{array}{c} X=[X_{1},X_{2},\cdots ,X_{n}] \end{array}$$ (1)

**Fig 2:**
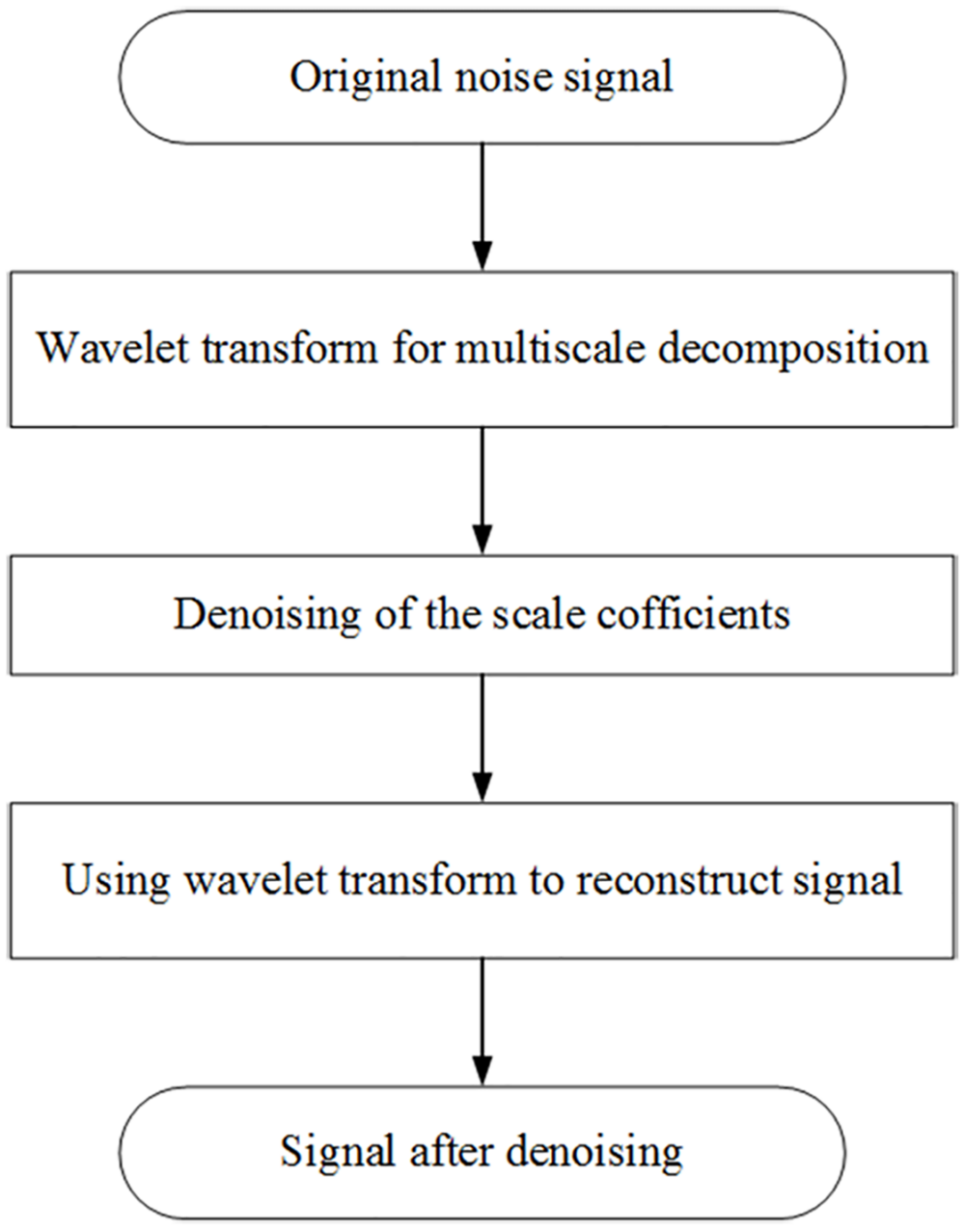
Flow chart of wavelet denoising. The principle is that the wavelet coefficients of the original signal are processed by using the threshold value function. The threshold function reflects whether the signal is above or below the threshold of the wavelet coefficients with respect to different processing strategies, and according to different spacecraft electrical characteristic data, hard or soft threshold functions are used to obtain a better filtering denoising effect. The flow chart of wavelet denoising is shown as Fig 2.

In this paper, the threshold denoising method is used to address the noise signal. The principle is that the wavelet coefficients of the original signal are processed by using the threshold value function. The threshold function reflects whether the signal is above or below the threshold of the wavelet coefficients with respect to different processing strategies, and according to different spacecraft electrical characteristic data, hard or soft threshold functions are used to obtain a better filtering denoising effect. The flow chart of wavelet denoising is shown as Fig 2.

Set *ω* to be the wavelet coefficient, where T is a given threshold, sign(*) is the symbol function, and the common threshold function is as follows: The hard threshold function of the electric characteristic signal of the spacecraft is as follows:

$$\begin{array}{c} W_{\text{REW}}=\left\{\begin{array}{cc} \omega & \;\left|\omega \right|\geq T\\ 0 & \;\left|\omega \right|<T \end{array}\right. \end{array}$$ (2)

The hard threshold function of the electric characteristic signal of the spacecraft is as follows:

$$W_{REW}=\{\begin{array}{ll} sign\left(\omega \right)\left(\omega -T\right) & \left|\omega \right|\geq T\\ 0 & \left|\omega \right|<T \end{array}$$ (3)

### B. Stacked auto-encoder

An auto-encoder, also known as auto-associator, is composed of two-layer neural network which has a hidden layer. The basic idea of auto-encoder is as follows. Encode the original signal and then reconstruction the signal. The weights between the two layers can be calculated by minimizing the reconstruction error between the input and output value of the network.

The equations of the encoder and decoder are as follows:

$$\begin{array}{c} Q_{m}=W_{mn}*X_{n}+B \end{array}$$ (4)

$$\begin{array}{c} Y_{n}=W1_{mn}*O_{m}+B1 \end{array}$$ (5)

where m is the number of hidden layer, n is the training samples’ dimension.

The error between the training samples and the reconstructed samples is selected as the cost function.

$$\begin{array}{c} error=\sum_{i=1}^{n}\left|X_{i}-Y_{i}\right| \end{array}$$ (6)

Then the gradient descent method can be used to minimize it and get the final initial weights of the stacked auto-encoder neural network.

**Fig 3:**
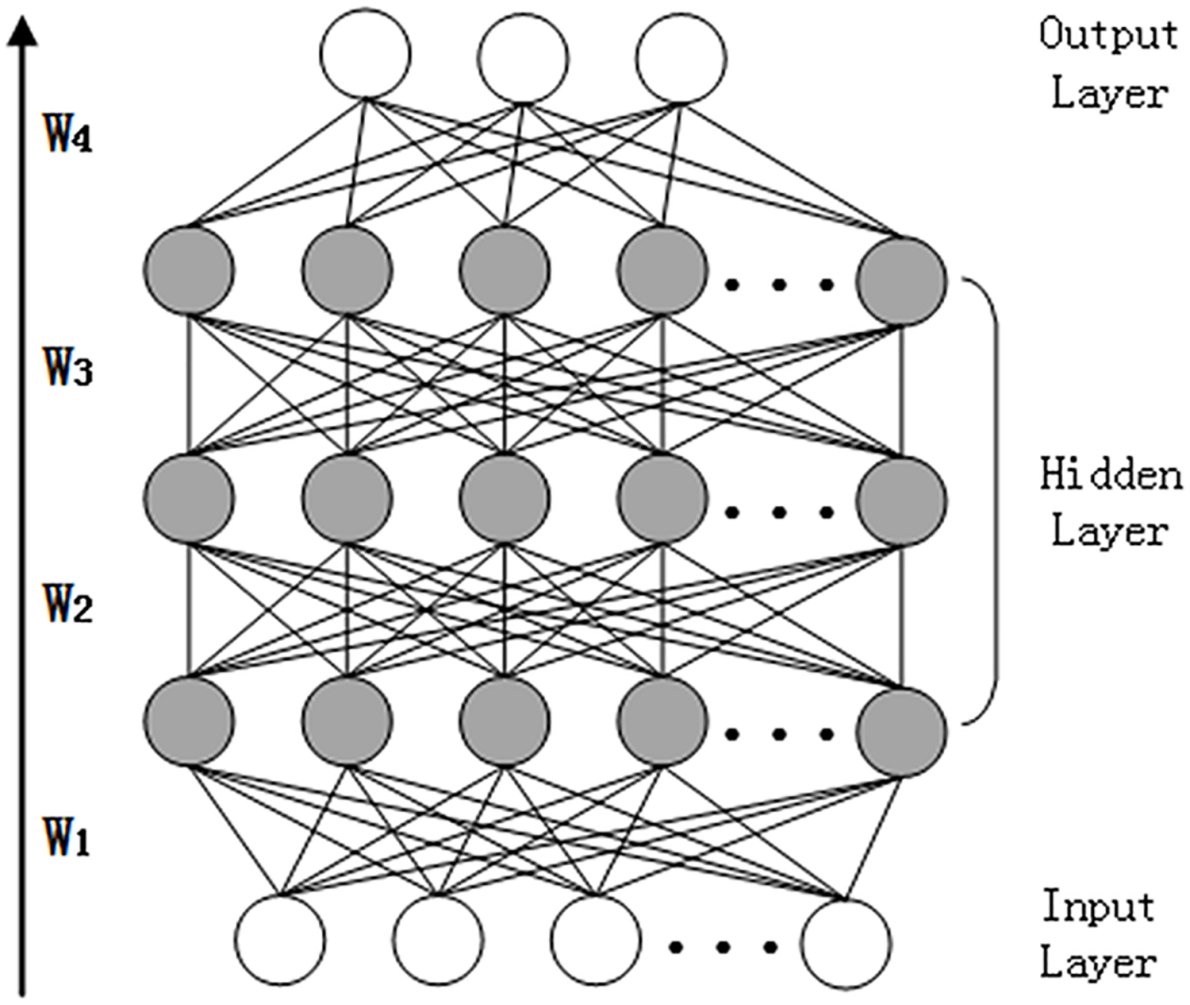
The topology of the SAE network. The representation ability of only auto-encoder is too limited. Thus the stacked auto-encoder composed of multiple auto-encoder is able to greatly improve the representational power. The activation value of one auto-encoder is the input of the upper auto-encoder. It can be used as a pre-training technique and it should belong to the unsupervised learning method for it doesn’t need label information at all. The topological graph of the SAE is shown in Fig 3.

The representation ability of only auto-encoder is too limited. Thus the stacked auto-encoder composed of multiple auto-encoder is able to greatly improve the representational power. The activation value of one auto-encoder is the input of the upper auto-encoder. It can be used as a pre-training technique and it should belong to the unsupervised learning method for it doesn’t need label information at all. The topological graph of the SAE is shown in Fig 3.

### C. Deep belief network

**Fig 4:**
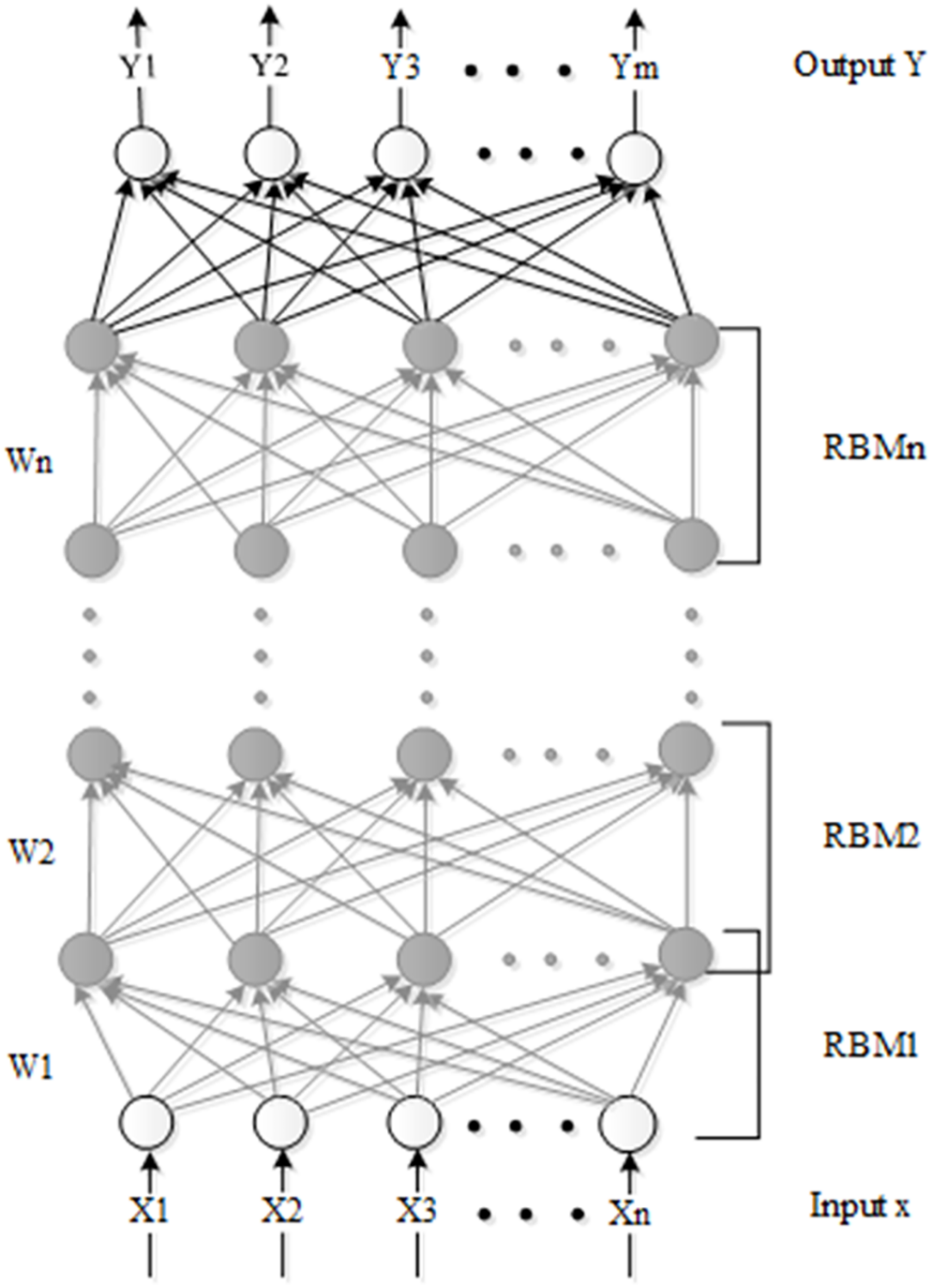
The topology of DBN network used in our article. The DBN network can not only solve a complex nonlinear problem but also extract more features from high-dimensional data. The DBN network is composed of an input layer, hidden layers, and an output layer. The DBN structure that is used in our article is presented in Fig 4.

Usually, DBN has more hidden layers than the BP network, and the network parameters of the DBN are initialized layer by layer [16, 17]. The DBN network can not only solve a complex nonlinear problem but also extract more features from high-dimensional data. The DBN network is composed of an input layer, hidden layers, and an output layer. The DBN structure that is used in our article is presented in Fig 4.

**Fig 5:**
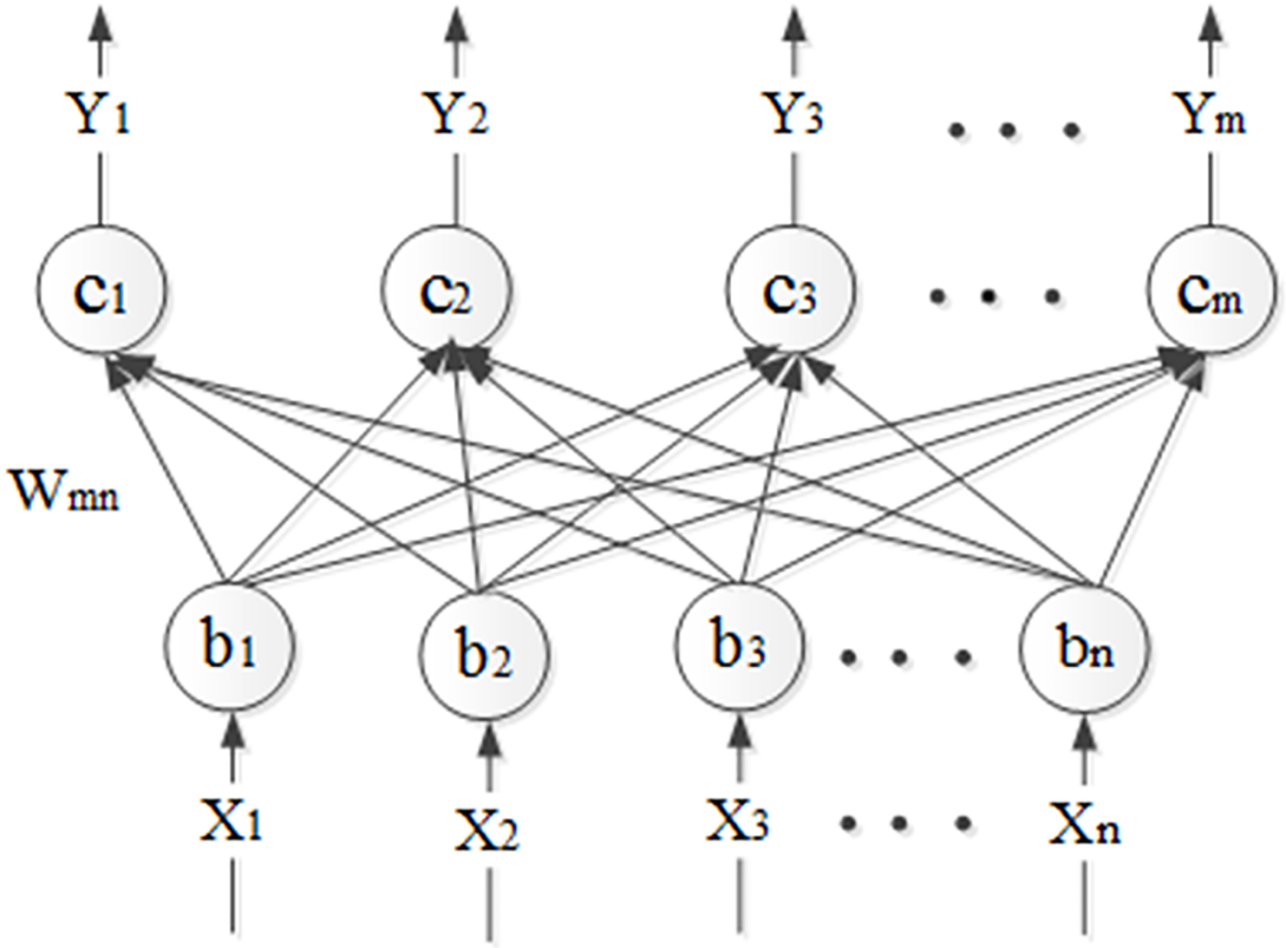
Network topology of Restricted Boltzmann machine. The RBM network consists of two layers, namely, the visible layer and the hidden layer. The neurons of the two layers are fully connected, while the neurons in the same layer are independent of one another. The two layers satisfy the Boltzmann distribution law. The network topology of the RBM is shown in Fig 5.

The deep belief network (DBN) is stacked by multiple Restricted Boltzmann machine (RBM) networks, and the output of the last RBM network is the next input [18, 19]. The RBM network consists of two layers, namely, the visible layer and the hidden layer. The neurons of the two layers are fully connected, while the neurons in the same layer are independent of one another. The two layers satisfy the Boltzmann distribution law. The network topology of the RBM is shown in Fig 5.

The activation function of the hidden layers is generally selected to be the sigmoid function, as follows:

$$\begin{array}{c} f\left(x\right)=\frac{1}{1+e^{-x}} \end{array}$$ (7)

Three parameters (*i*.*e*.,*W*_*mn*_, *B*_*n*_ *and* *C*_*m*_) must be obtained by training the DBN network. The weights of the network and the offsets *B*_*n*_ and *C*_*m*_ are defined as follows:

$$\begin{array}{c} W_{mn}=\left[\begin{array}{cccc} W_{11} & W_{12} & \cdots & W_{1n}\\ W_{21} & W_{22} & \cdots & W_{2n}\\ \vdots & \vdots & \vdots & \vdots \\ W_{n1} & W_{n2} & \cdots & W_{nn} \end{array}\right]\; \end{array}$$ (8)

$$\begin{array}{c} B_{n}=\left[b_{1},b_{2},...b_{n}\right],C_{m}=\left[c_{1},c_{2},...c_{n}\right] \end{array}$$ (9)

The energy function is given as follows:

$$\text{E}\left(\text{x},\text{y},\theta \right)=-\underset{ij}{\sum }w_{ij}x_{ij}y_{ij}-\underset{i}{\sum }b_{i}x_{i}-\underset{j}{\sum }c_{j}y_{j}$$ (10)

$$\begin{array}{c} \theta =\{\omega ,b,c\} \end{array}$$ (11)

where *θ* is a given model parameter, *w*_*ij*_ represents the association weights between the visual and hidden nodes. *b*_*i*_ is the node offset of the visual layer, i is the numeric index of the nodes in the visual layer, *c*_*j*_ is the node offset of the hidden layer, and j is the numeric index of the nodes in the visual layer.

### D. Random forest algorithm

Random forest is an ensemble classifier that is composed of a group of decision trees{*h*(*X*,*θ*_*k*_),*k* = 1, 2, ⋯, *K*}, where {*θ*_*k*_} is subject to independent and identically distributed random vectors, and K is the number of decision trees. In a given electrical signal variable X, each decision tree classifier votes to determine the optimal classification result [20, 21, 22]. The steps to generate the random forest are as follows:

1. From the original electrical signal training data, using the method of bootstrap, select K new independent sample sets randomly and construct K decision trees. The sample comprises K out-of-bag data, which is not selected.
2. Assume N characteristics, and then, select characteristics randomly (*m*_*try* ≤ *N*_). By calculating the amount of information that is contained in each feature, we select the characteristic that has the best classification ability to perform node splitting.
3. Each tree grows to a maximum and does not perform any cutting.

**Fig 6:**
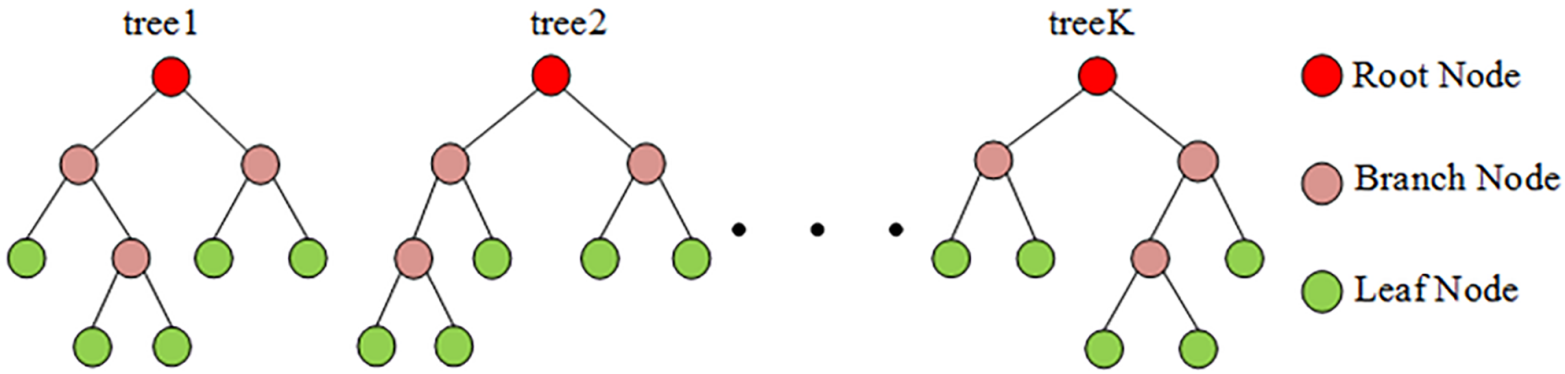
The structure of decision trees. Fig 6 shows K decision trees, which consist of the root node, branch node, and leaf node. The root node shows the starting point for the classification, which represents the most appropriate electrical signal feature of the decision tree. The branch nodes divide the data into two clusters that have different rules. The leaf nodes obtain the electrical data classification results.

**Fig 7:**
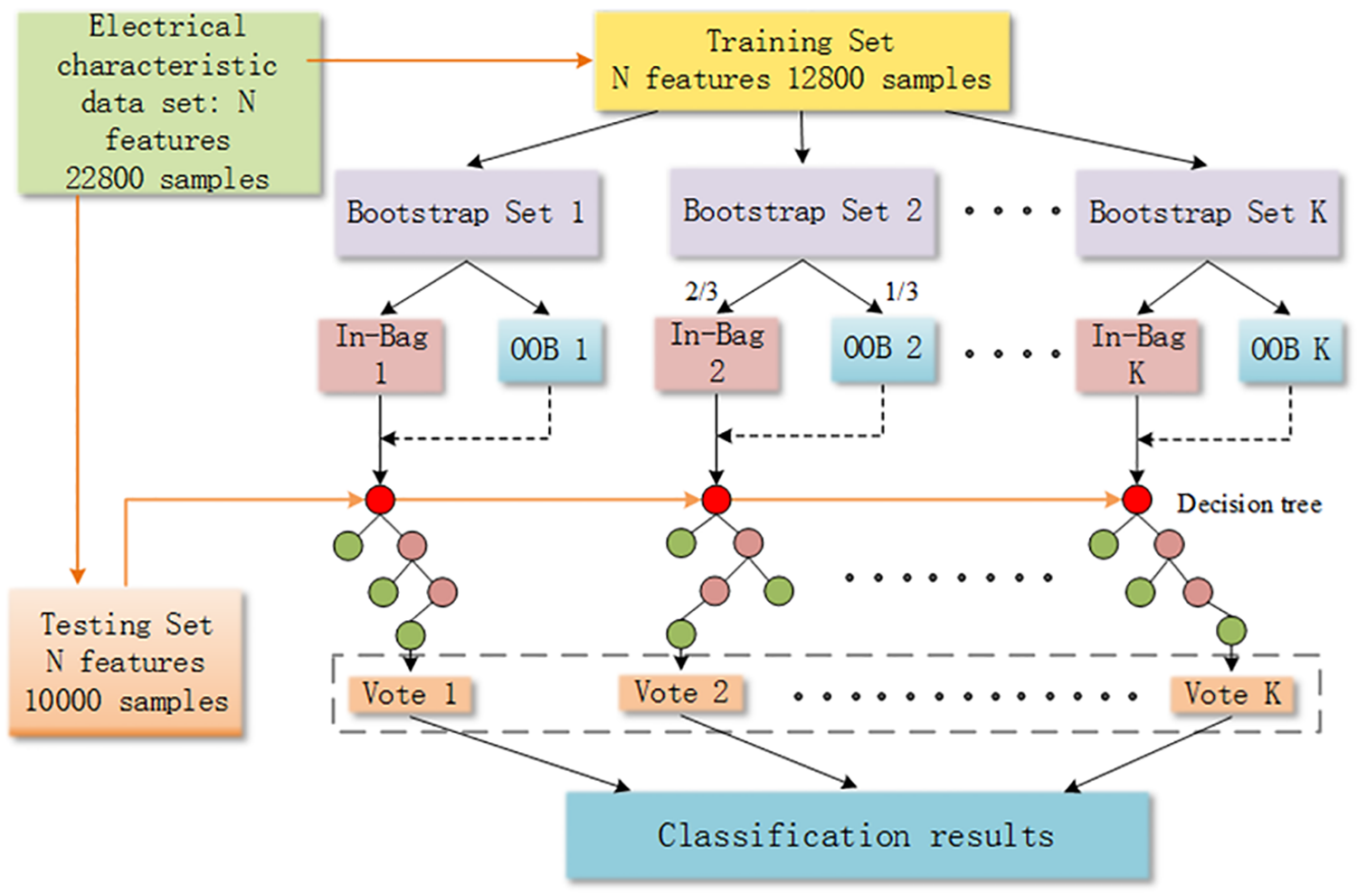
The structure of random forest algorithm. The random forest is composed of the generated trees, and we use the random forest to classify the electrical signal test data. The classification result is decided by the decision trees. The structure of the random forest algorithm used in our article is shown in Fig 7.

Fig 6 shows K decision trees, which consist of the root node, branch node, and leaf node. The root node shows the starting point for the classification, which represents the most appropriate electrical signal feature of the decision tree. The branch nodes divide the data into two clusters that have different rules. The leaf nodes obtain the electrical data classification results. The structure is shown as follows:

The random forest is composed of the generated trees, and we use the random forest to classify the electrical signal test data. The classification result is decided by the decision trees. The structure of the random forest algorithm used in our article is shown in Fig 7.

Given a set of classifiers *h*_1_(*X*), *h*_2_(*X*) ⋯, *h*_*k*_(*X*) the training set of each classifier comes from the original data (*X*, *Y*), which is subject to random distribution. The margin function is defined as

$$\begin{array}{c} \text{mg}\left(X,Y\right)=av_{k}I\left(h_{k}\left(X\right)=Y\right)-\underset{j\neq Y}{max\;}av_{k}I\left(h_{k}\left(X\right)=j\right) \end{array}$$ (12)

where *I*(⋅) is the indication function, Y is the correct classification vector, j is the false classification vector, and *av*_*k*_(⋅) represents the average.

The margin function is used to measure the degree of the average correct classification and the average error classification. The greater the margin value is, the more reliable the classification prediction.

The generalization error is defined as

$$\begin{array}{c} {\text{PE}}^{*}=P_{X,Y}\left(mg\left(X,Y\right)<0\right) \end{array}$$ (13)

In the formula, subscripts X, Y represent that the probability P covers X, Y space.

With an increase in the number of decision trees in the random forest, all of the sequences of *θ*_1_, *θ*_2_, ⋯, *θ*_*k*_, PE*(*θ*_*k*_ is an independent and identically distributed random variable) converge to the following:

$$\begin{array}{c} P_{X,Y}\{P_{\theta }\left(h\left(X,\theta \right)=Y\right)-maxP_{\theta }\left(h_{j\neq Y}^{\left(X,\theta \right)}=j\right)<0\} \end{array}$$ (14)

This formula indicates that the random forest will not produce an over-fitting problem when there is an increase in the decision trees, but it could produce a certain degree of generalization error [23, 24].

## IV. Experimental results

### A. Experiment data presentation

**Fig 8:**
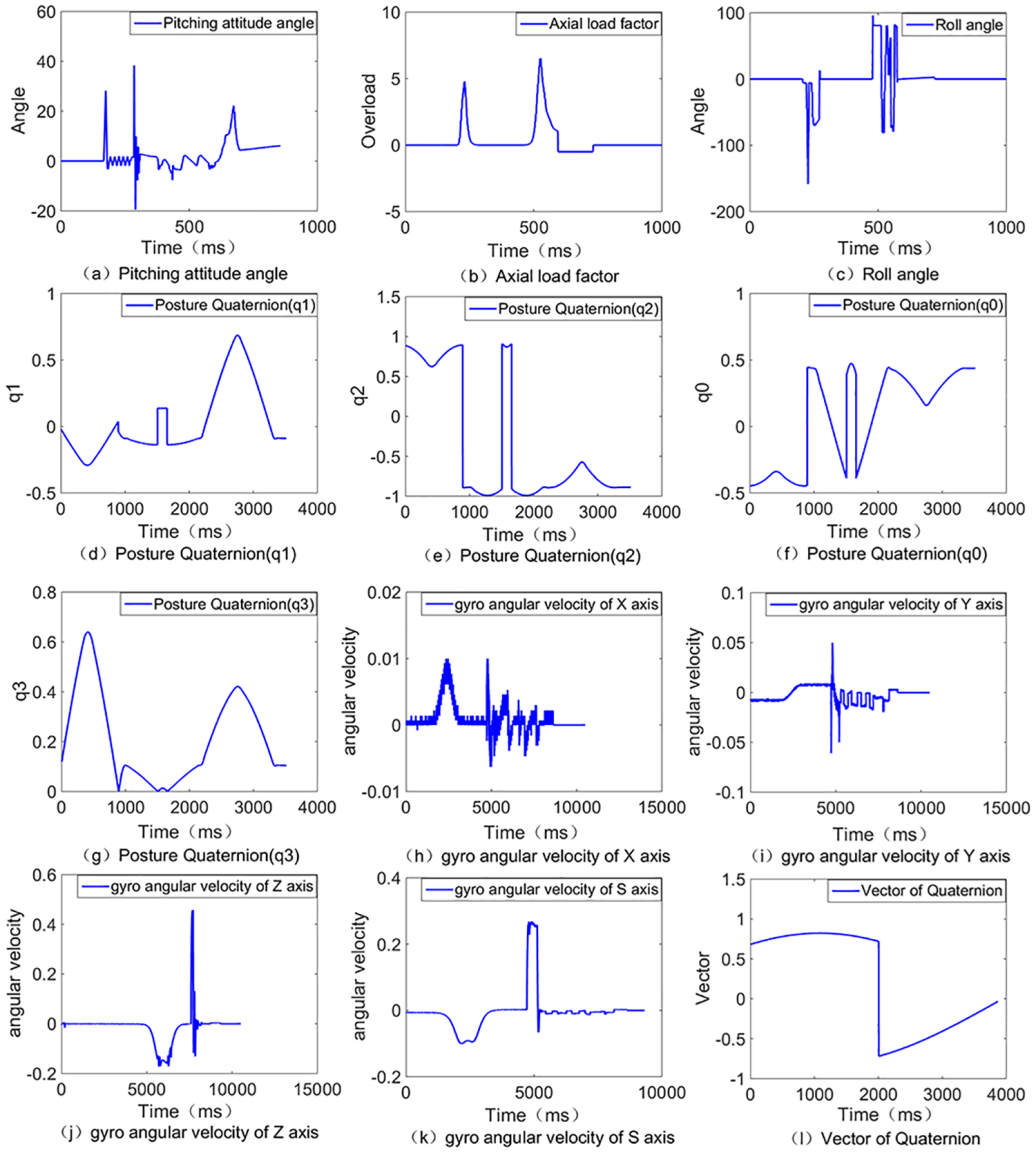
Physical meaning of some electrical properties data. There are 19 different types of signals, and sample labels from 13 modes in the spacecraft flying data are presented in this study. A total of 22800 samples were acquired, where each sample contained 1000 features. More specifically, the physical meaning of the data is shown in Fig 8, combining twelve elements of the physical process: (a)Pitching attitude angle, (b)Axial load factor, (c)Roll angle, (d)Posture Quaternion(q1), (e)Posture Quaternion(q2), (f)Posture Quaternion(q0), (g)Posture Quaternion(q3), (h)Gyro angular velocity of X axis, (i)Gyro angular velocity of Y axis, (j)Gyro angular velocity of Z axis, (k) Gyro angular velocity of S axis, (k) Vector of Quaternion.

The experimental data comes from typical electrical characteristics data of spacecraft electronic load systems. In the process of spacecraft load testing, the electronic load bus of the spacecraft is monitored by the electric characteristic monitoring platform, and it records the original data. There are 19 different types of signals, and sample labels from 13 modes in the spacecraft flying data are presented in this study. A total of 22800 samples were acquired, where each sample contained 1000 features. More specifically, the physical meaning of the data is shown in Fig 8.

The data set is firstly divided into two different sets, which are referred to as the training set and testing set before classification. Namely, 12800 original signals that comprised 56% of the total were used for model training, and 10000 original signals that comprised 44% of the total were used for testing the performance of the training model. The original data were normalized before the training model was built. The classification model was then trained by using the training data set, and the testing data set was applied for model validation.

### B. Number of decision trees selection

**Fig 9:**
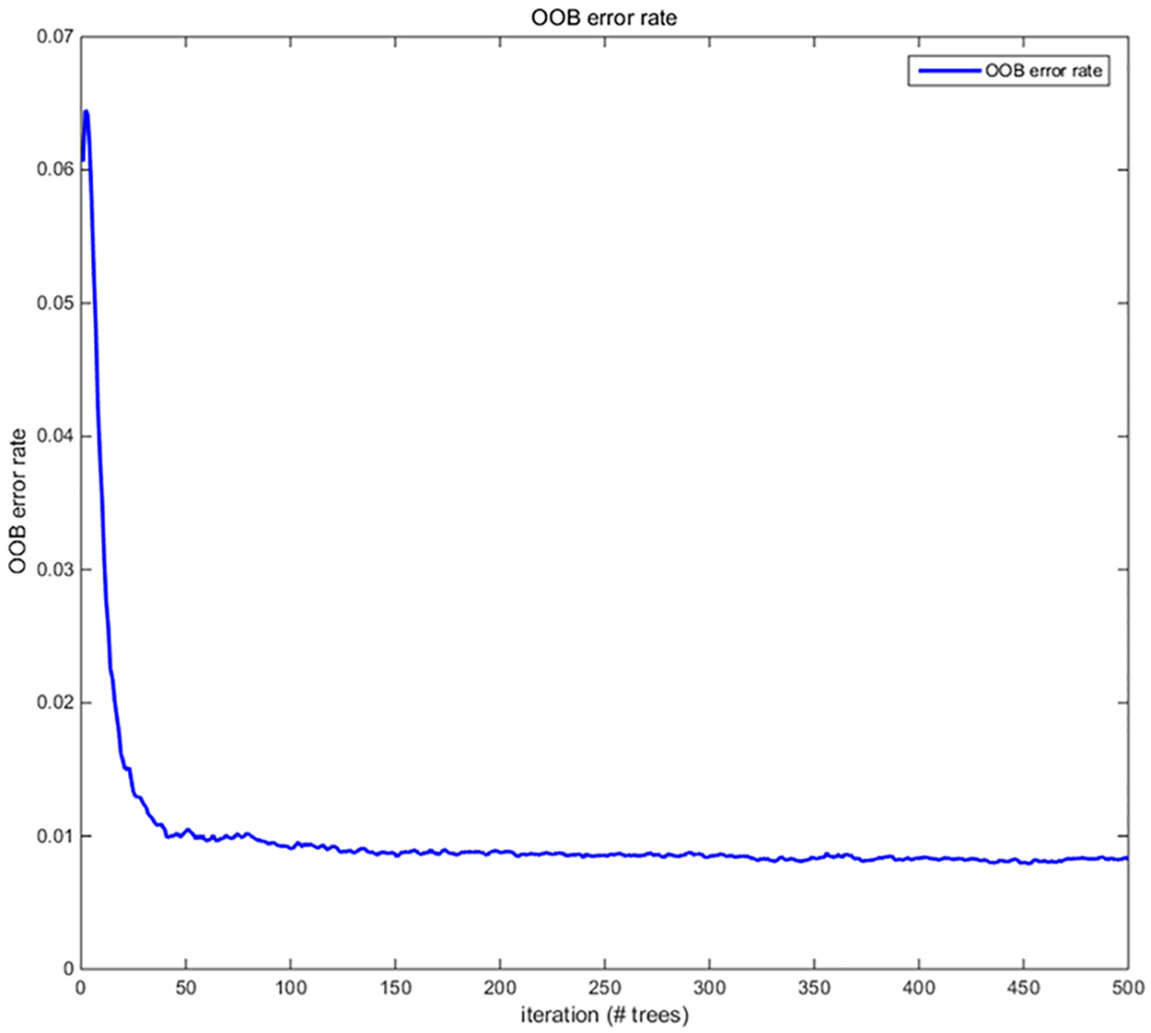
Number of decision trees and OOB error rate curve. Fig 9 shows that the classification error rate decreases with an increase in the number of decision trees. After reaching the 100 trees, the classification error trends to be stable (approximately 0.01).

Fig 9 shows that the classification error rate decreases with an increase in the number of decision trees. After reaching the 100 trees, the classification error trends to be stable (approximately 0.01). However, the training time of the model increases with the increase in the number of decision trees. Therefore, considering the problem of time complexity, we selected 100 decision trees that comprised the random forest, to classify and predict the electrical signal sample sets. This approach not only ensures the accuracy of the classification but also shortens the time needed for training and classification.

### C. Comparison of different algorithms

The classification accuracy and classification time are the important symbols of the evaluation algorithm. In this paper, we use different algorithms, including Naive Bayesian Model, K-Nearest Neighbour, Support Vector Machine, and random forest, to classify the spacecraft electrical signal data, and we obtain the classification results of the different algorithms. At the same time, PCA and DBN are used for feature extraction, and then, we classify and recognize the electrical data. Finally, we compare the performance of different algorithms before and after feature extraction. By comparison, it is found that when the feature dimension is too large, the classification accuracy will be reduced because of the curse of dimensionality. The reduction of the sample dimension can not only improve the calculation speed, but also improve the classification accuracy of the signal.

**Fig 10:**
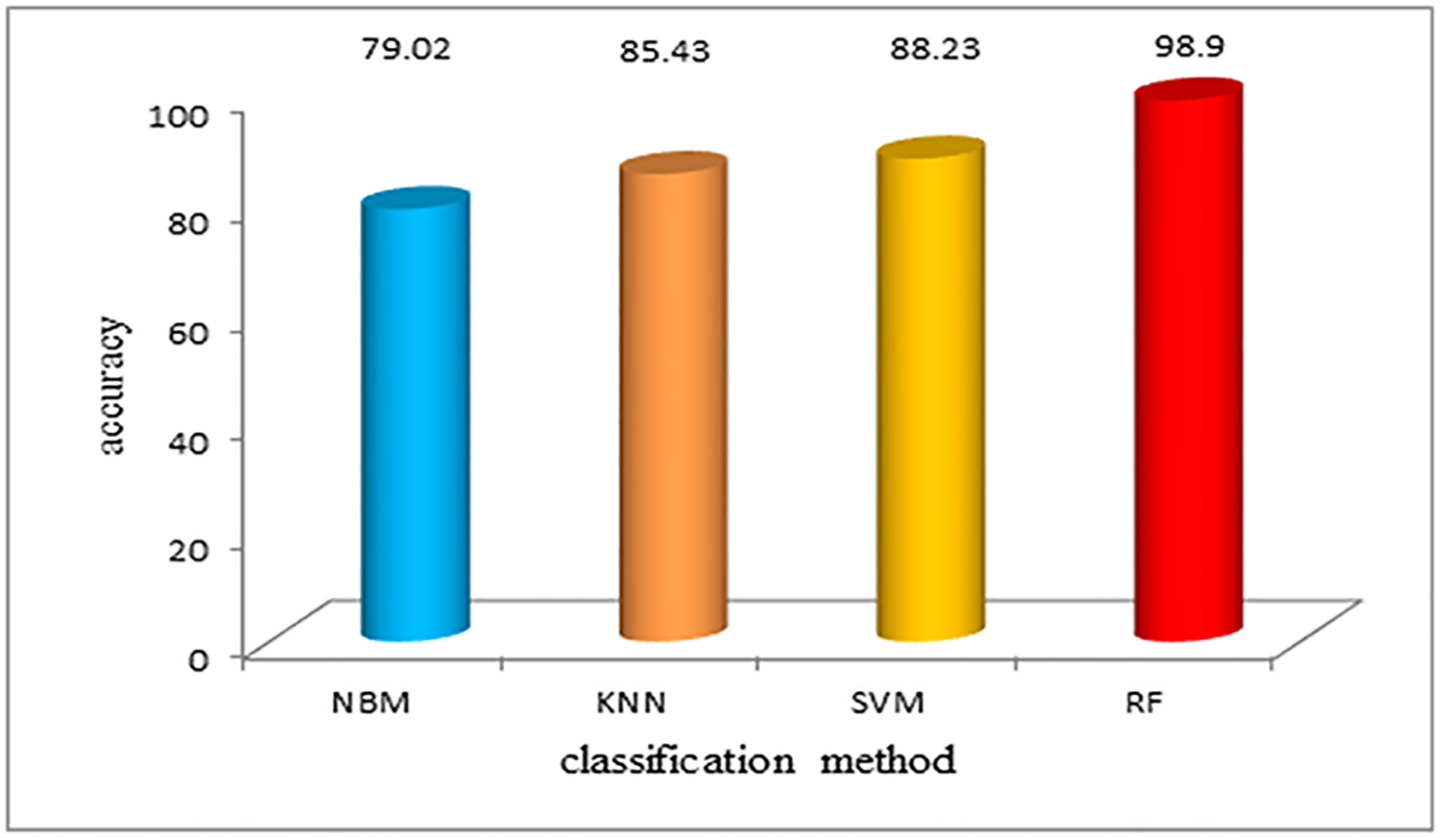
Classification accuracy using different algorithms before dimension reduction. Before the dimension reduction of the data, the classification accuracy of the four algorithms is shown in Fig 10. Comparing the accuracy of the algorithm, the RF algorithm has the highest classification accuracy. Due to the limitation of the multi-class classification problems, random forest has better tolerance, excellent performance, and the highest classification accuracy before dimension reduction.

We randomly selected 50% sample data as training set, the other 50% samples as a test set, the algorithm model is trained on the training set and test set for classification. Before the dimension reduction of the data, the classification accuracy of the four algorithms is shown in Fig 10. Comparing the accuracy of the algorithm, the RF algorithm has the highest classification accuracy. Due to the limitation of the multi-class classification problems, random forest has better tolerance, excellent performance, and the highest classification accuracy before dimension reduction.

**Fig 11:**
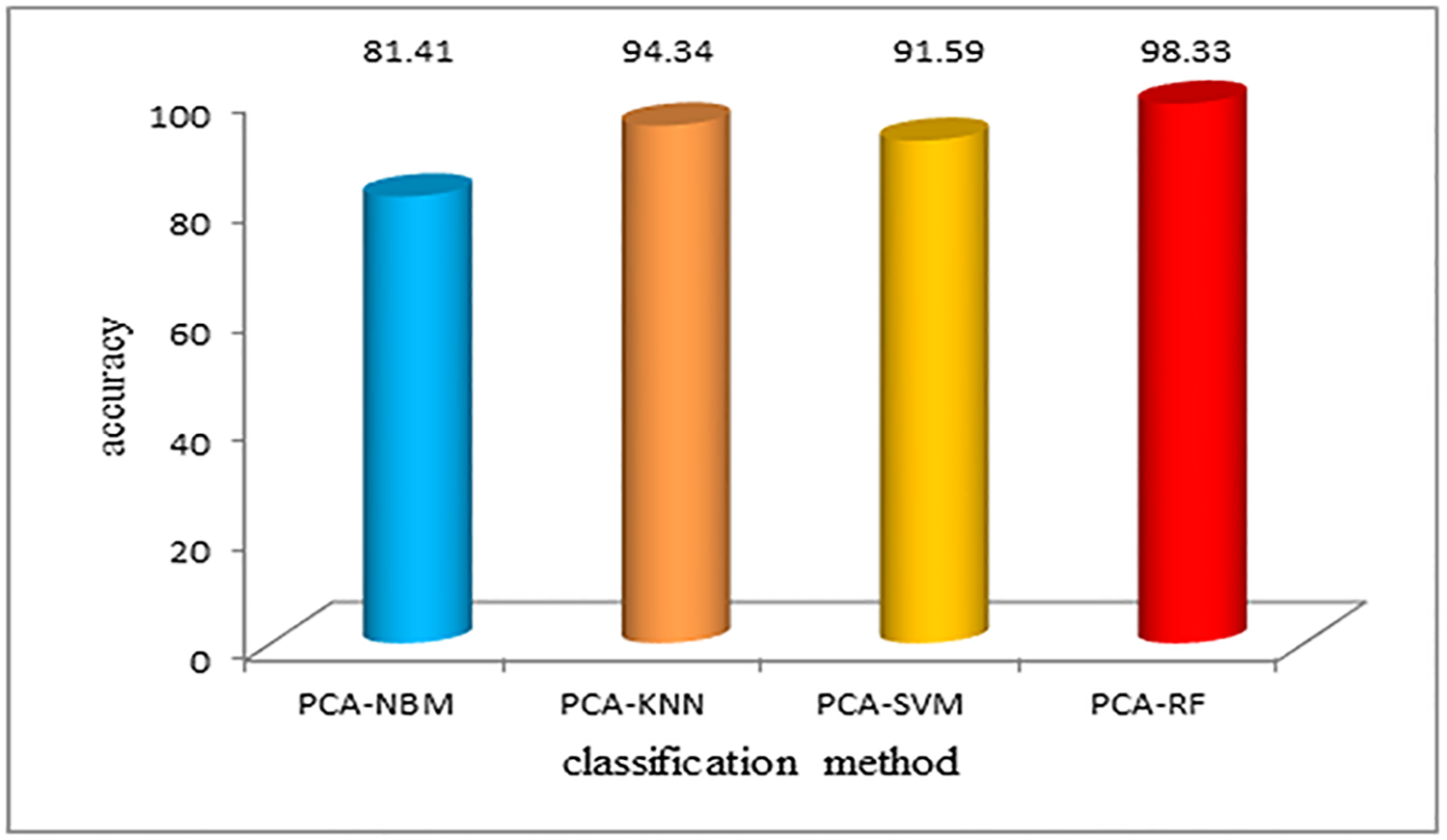
Classification accuracy using different algorithms after PCA dimension reduction. After using the PCA method to reduce the dimension of the data, the classification accuracy was improved, and the PCA-RF algorithm still showed excellent performance, as shown in Fig 11.

After using the PCA method to reduce the dimension of the data, the classification accuracy was improved, and the PCA-RF algorithm still showed excellent performance, as shown in Fig 11.

**Fig 12:**
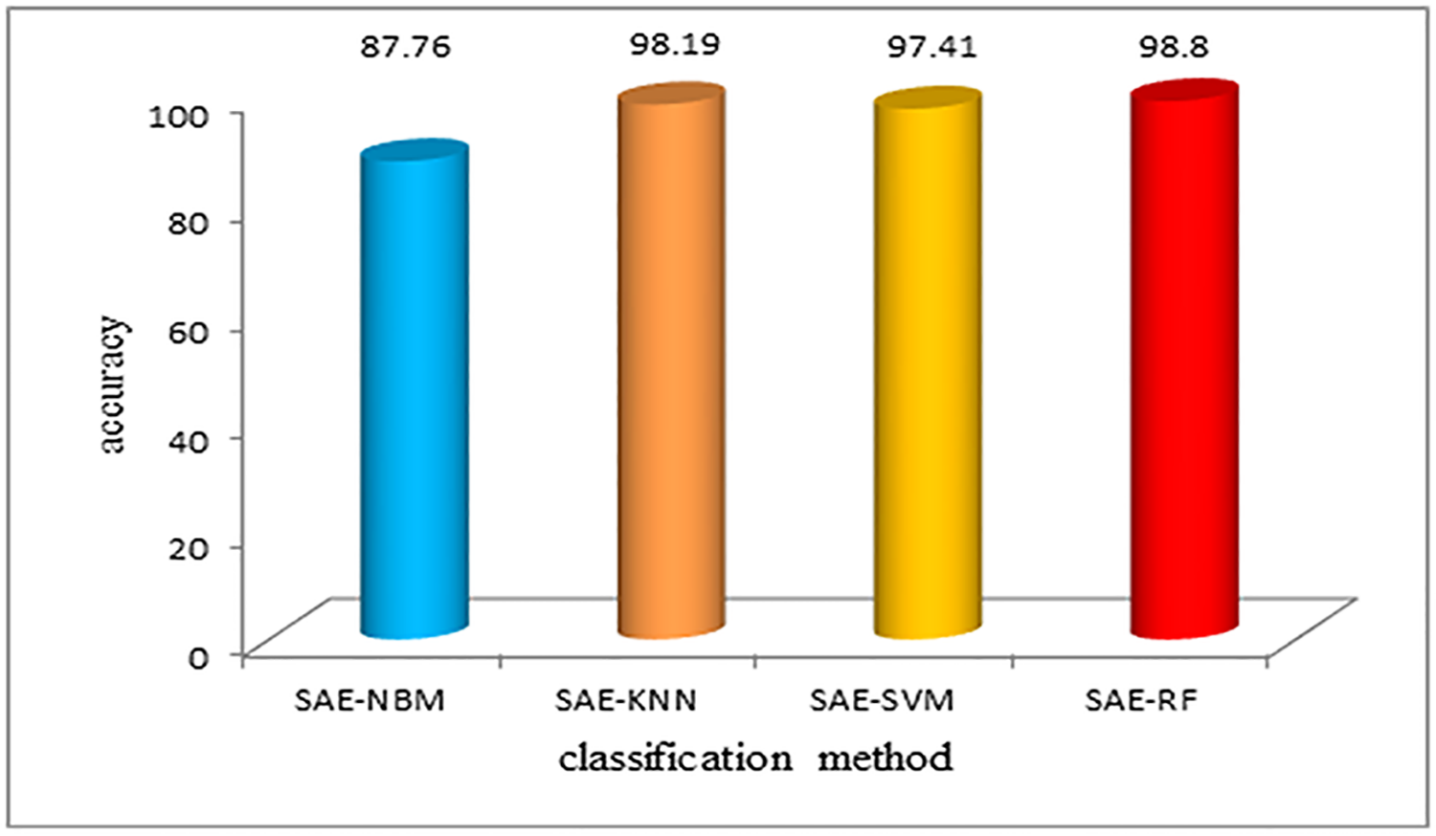
Classification accuracy using different algorithms after SAE dimension reduction. Then SAE was applied into the data dimension reduction, the accuracy of the classification result has been further improved, which is shown in Fig 12. And the SAE-RF algorithm improved almost 0.5% compared with PCA-RF.

Then SAE was applied into the data dimension reduction, the accuracy of the classification result has been further improved, which is shown in Fig 12. And the SAE-RF algorithm improved almost 0.5% compared with PCA-RF.

**Fig 13:**
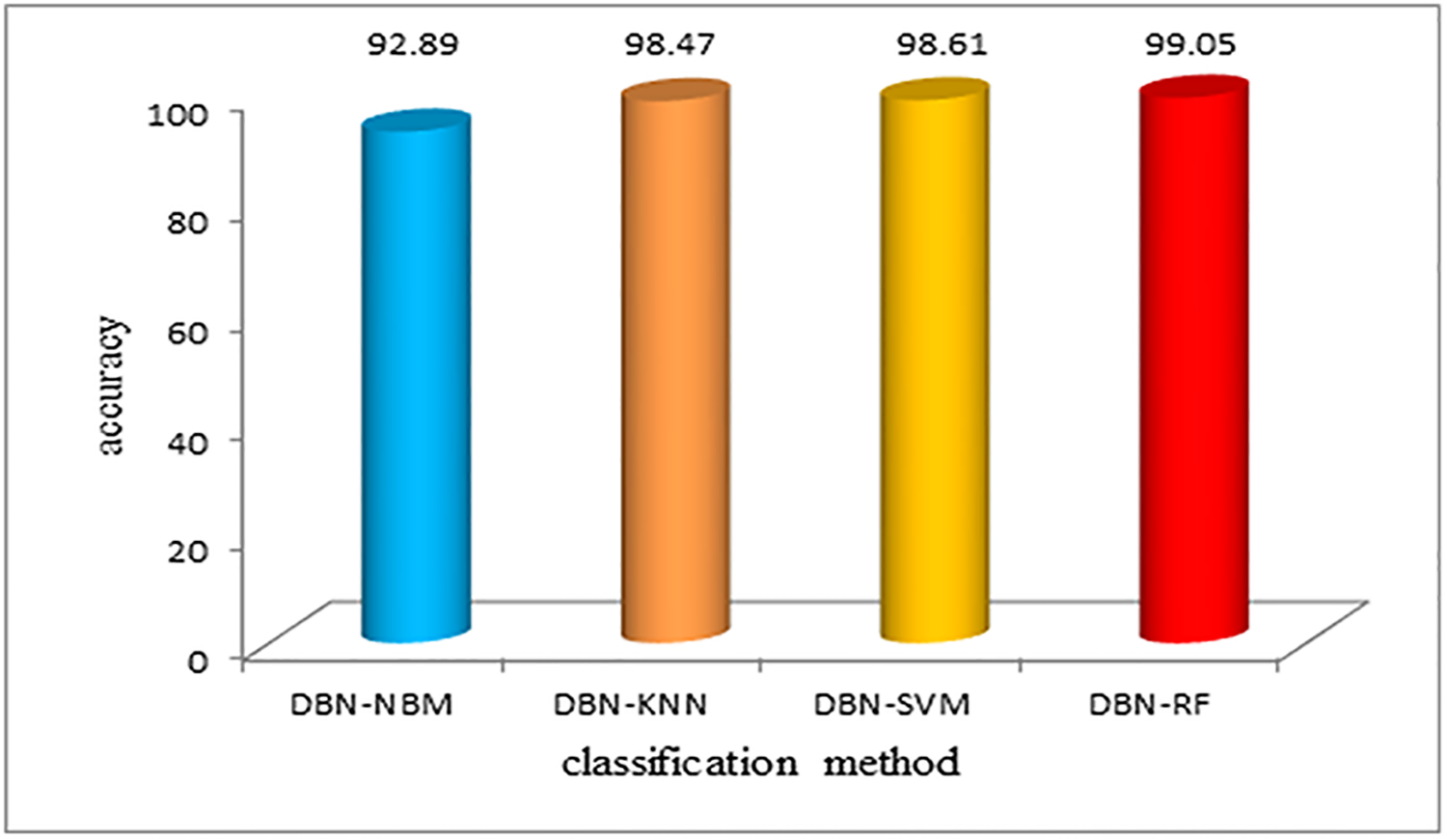
Classification accuracy using different algorithms after DBN dimension reduction. When we used the DBN method to reduce the dimension of the data, the classification performance of the four algorithms was greatly improved, which is shown in Fig 13. Most notably, the performance of the DBN-RF algorithm is especially prominent.

When we used the DBN method to reduce the dimension of the data, the classification performance of the four algorithms was greatly improved, which is shown in Fig 13. Most notably, the performance of the DBN-RF algorithm is especially prominent.

We compared different algorithms performances before and after feature extraction on the data. When the feature dimension of the samples is too large, the classification accuracy will be low due to the dimensionality disaster. Performing dimension reduction on the samples can not only improve the computational speed but also improve the classification accuracy. Table 1 shows the recognition results of the different algorithms used in this paper in which the training sample is 20%, the test sample is 80%, given the classification results of different algorithms clearly and intuitively. Table 2 shows the recognition results of the different algorithms, in which training sample is 30% and test sample is 70%. At last, Table 3 shows the recognition results of the different algorithms, in which training sample is 40% and test sample is 60%.

**Table 1:** Comparison of the training time and forecasting accuracy (training 20%, testing 80%).

Method	Accuracy (%)	Training Time (sec)	Training Time (sec)
*NBM*	71.91	89.09	3117.25
*KNN*	77.84	–	85.2
*SVM*	88.23	271.84	113.24
*RF*	98.9	189.93	0.6
*PCA*–*NBM*	78.74	8.34	308.22
*PCA*–*KNN*	92.46	–	7.17
*PCA*–*SVM*	85.82	5.91	6.85
*PCA*–*RF*	94.14	4.81	0.17
*SAE*–*NBM*	91.1	9.51	397.92
*SAE*–*KNN*	98.38	–	9.96
*SAE*–*SVM*	98.59	4.07	2.51
*SAE*–*RF*	98.83	4.49	0.12
*DBN*–*NBM*	85.04	9.85	400.59
*DBN*–*KNN*	98.41	–	9.47
*DBN*–*SVM*	98.44	2.34	2.02
**DBN-RF**	**98.76**	**4.08**	**0.12**

**Table 2:** Comparison of the training time and forecasting accuracy (training 30%, testing 70%).

Method	Accuracy (%)	Training Time (sec)	Training Time (sec)
*NBM*	76.11	91.76	3014.45
*KNN*	79.44	–	111.39
*SVM*	83.11	604.65	143.27
*RF*	98.76	98.72	0.31
*PCA*–*NBM*	7.22	290.2	78.78
*PCA*–*KNN*	94.06	–	9.67
*PCA*–*SVM*	86.42	11.26	9.69
*PCA*–*RF*	94.22	8.35	0.11
*SAE*–*NBM*	91.51	10.07	320.57
*SAE*–*KNN*	98.63	–	12.95
*SAE*–*SVM*	98.8	6.95	2.75
*SAE*–*RF*	98.73	8.48	0.11
*DBN*–*NBM*	86.72	9.69	320.57
*DBN*–*KNN*	98.48	–	12.85
*DBN*–*SVM*	98.57	3.82	2.19
**DBN-RF**	**98.93**	**4.08**	**0.12**

**Table 3:** Comparison of the training time and forecasting accuracy (training 40%, testing 60%).

Method	Accuracy (%)	Training Time (sec)	Training Time (sec)
*NBM*	75.58	103.68	2949.65
*KNN*	81.7	–	124.3
*SVM*	86.61	1064.51	161.45
*RF*	99.06	148.05	0.27
*PCA*–*NBM*	7.38	269.58	78.91
*PCA*–*KNN*	93.73	–	11.18
*PCA*–*SVM*	87.46	17.73	11.39
*PCA*–*RF*	94.51	12.14	0.09
*SAE*–*NBM*	91.92	9.56	330.59
*SAE*–*KNN*	98.72	–	14.92
*SAE*–*SVM*	98.88	10.74	2.67
*SAE*–*RF*	99.12	12.49	0.09
*DBN*–*NBM*	86.87	11.56	311.805
*DBN*–*KNN*	98.65	–	14.89
*DBN*–*SVM*	98.54	5.52	2.15
**DBN-RF**	**99.13**	**11.73**	**0.27**

Table 1 shows that before feature extraction, the classification accuracy of the NBM algorithm and KNN algorithm are 79.02% and 85%, respectively. The classification accuracy of the SVM algorithm is slightly higher. The random forest algorithm has the highest accuracy rate of 98.9%, and it has better performance compared to the other algorithms, which is also fast. After the feature extraction, the data dimension, and the computational complexity are reduced, which makes the calculation speed and accuracy improve significantly. Both before and after the feature extraction, the random forest algorithm shows excellent classification performance. The training time is short, and the accuracy is guaranteed.

**Fig 14:**
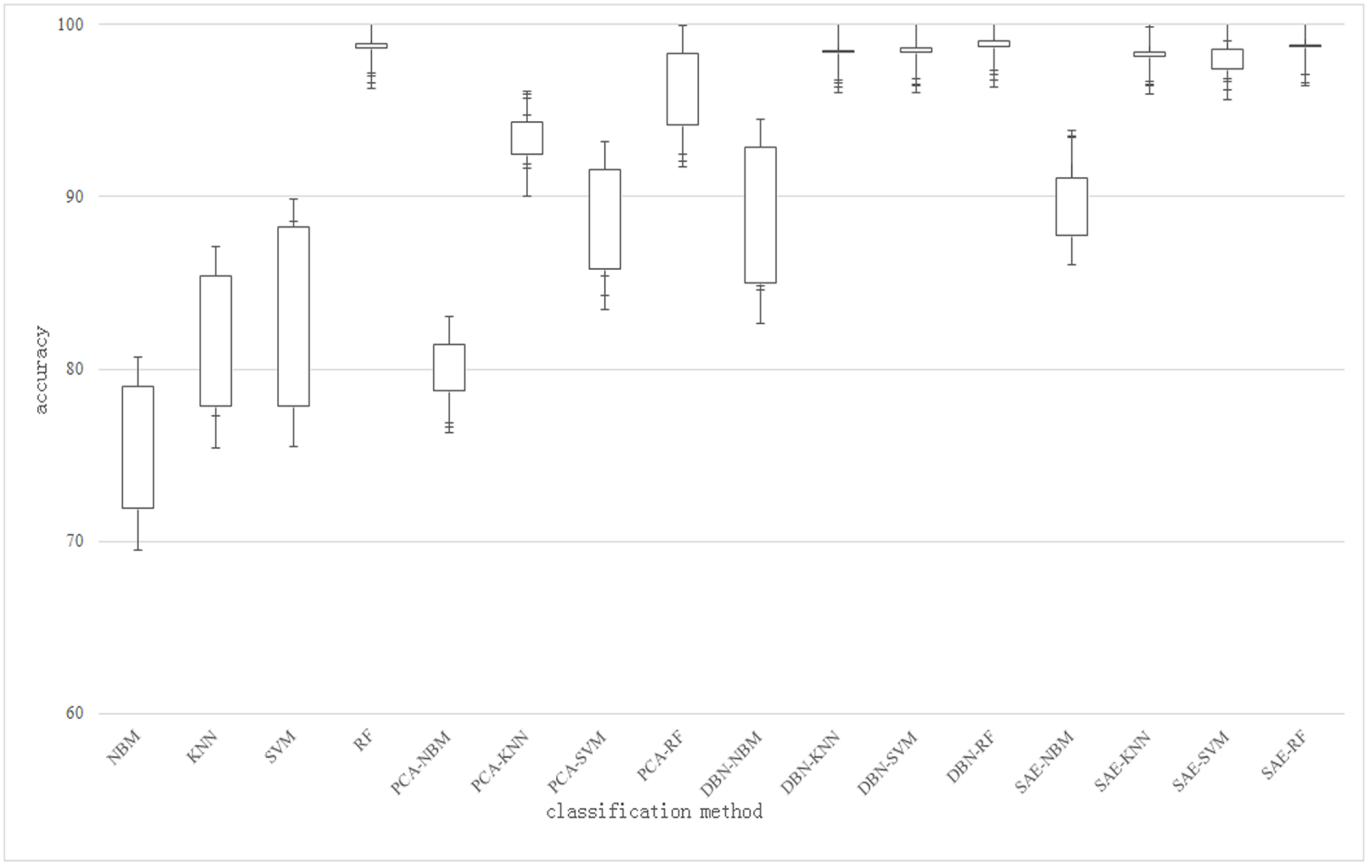
The statistics of the different algorithms. For a more intuitive representation of the classification algorithm, the box shaped figure of classification algorithm model of accuracy is as shown in Fig 14, which is the statistics of four groups of different number of training set and test set showing the accuracy of classification which is before and after using dimension reduction. In Fig 14, we also can directly see the random forest algorithm has excellent classification performance, compared to other algorithms.

In contrast to Tables 1, 2 and 3, the data after dimensionality reduction has a significant improvement in the speed of model building and classification than that of dimensionality reduction. Although the SVM algorithm is slightly faster than the random forest algorithm in some individual models, random forest algorithm has maintained a very excellent performance for the prediction speed of unknown samples. So in contrast to the verification process repeatedly, random forest has the performance which is more suitable and basically meet ting the needs of practical application in the process of electronic load signals of spacecraft power system. For a more intuitive representation of the classification algorithm, the box shaped figure of classification algorithm model of accuracy is as shown in Fig 14, which is the statistics of four groups of different number of training set and test set showing the accuracy of classification which is before and after using dimension reduction. In Fig 14, we also can directly see the random forest algorithm has excellent classification performance, compared to other algorithms.

**Fig 15:**
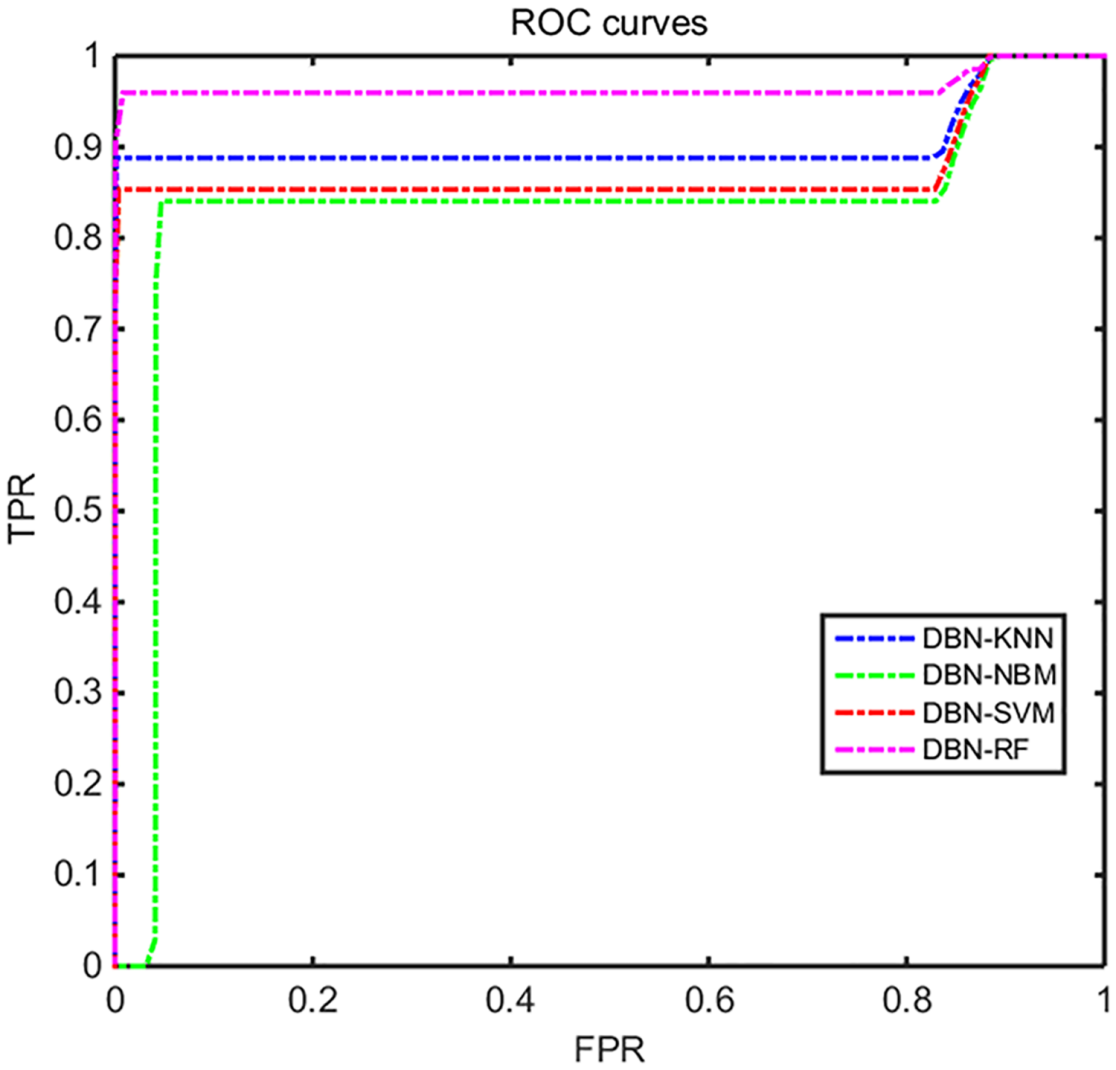
The ROC curve of different algorithm. We selected the classification results of a representative class, drew its ROC curve, and compare the classification performance of the different algorithms, which is shown in Fig 15. From Fig 15, we can obviously see that the classification performance of DBN-RF is better than that of the other algorithms that were used in this article. These results show that the method proposed in this article is effective for processing multi-class high-dimensional spacecraft electrical signals.

Receiver operating characteristic (ROC) curves are commonly used to present the results of binary decision problems in machine learning, which reflects the sensitivity and specificity of the comprehensive indicators of the continuous variables [25]. The sensitivity represents the true positive rate on the vertical coordinate, and the specificity represents the false positive rate on the horizontal. The performances of the algorithms are comparable in ROC space. The area under the curve (AUC) of the ROC reflects the ability to distinguish the events by the classification algorithm, and the greater the area under the curve is, the higher the diagnostic accuracy. After dimension reduction by DBN, the performance of each of the classification algorithms is greatly improved. Therefore, we selected the classification results of a representative class, drew its ROC curve, and compare the classification performance of the different algorithms, which is shown in Fig 15. From Fig 15, we can obviously see that the classification performance of DBN-RF is better than that of the other algorithms that were used in this article. These results show that the method proposed in this article is effective for processing multi-class high-dimensional spacecraft electrical signals.

## V. Conclusions

In this paper, a combination of the random forest algorithm and the data reduction method is proposed, and this combination can identify and classify well the multi-class electrical signals of spacecraft. The main feature of the original data is extracted by DBN in the process of electrical characteristic identification. Dimension reduction of the spacecraft electrical characteristic data, which has a high dimension, is realized. Then, we used the random forest algorithm to recognize the spacecraft electrical characteristic data. This approach not only reduces the time needed for the computation but also further enhances the performance of the classifier. The algorithm is a simulation experiment of original data from a certain spacecraft, which can be applied directly to the classification and identification of the spacecraft electrical signals. The experimental results show that the recognition method based on DBN-RF has a higher classification accuracy and better recognition efficiency. The random forest algorithm has many advantages in addressing the data, and it is very flexible and adaptive when addressing fuzzy data, which has specific rules. According to the algorithm of the model, the calculated complexity is still large. In future research, we can combine our proposed method with other dimension reduction methods and, then, test the effectiveness of the test methods on different data sets, followed by performing further optimization to construct a classifier that has even better performance.

## Supporting information

S1 Dataset Including the standard data, test data, simulation data and typical data. (RAR)
